# Parliament and the Professions

**Published:** 1919-08-16

**Authors:** 


### PARLIAMENT AND THE PROFESSIONS.

### Nurses and the War Medal.

Colonel Ward asked the Secretary of State for War if he would consider the desirability of awarding the General Service Medal to all those nurses and V.A.D. nurses who were engaged in nursing soldiers and sailors in home hospitals. Mr. Churchill replied that the question of recognition of home service is under consideration, but that every medal given to people who did not take part in the fighting detracts from distinction.

### Uncertifiable Mental Cases.

Colonel Wedgwood asked the Minister of Health what steps he is taking with a view to providing in all areas convalescent homes for the benefit of early uncertifiable mental cases, in order to prevent their growing worse and becoming certifiable and a burden on the State, and, in order that such homes shall not be regarded as half-way houses to asylums, would he ensure that intending patients should enter voluntarily, as they would a hospital, and that the homes so provided shall have no connection with lunacy administration. Major Astor replied that this point, together with others, was receiving the careful consideration of the Ministry, and it is hoped that suitable legislation, which would be necessary, may be shortly introduced.

### Hospital Ships in North Russia.

Mr. Churchill stated that two hospital ships are being used to evacuate patients from the military hospitals in North Russia. An adequate supply of doctors and hospital equipment are present at both Archangel and Murmansk. Six British nurses are doing duty in the hospital at the latter place, and none are stationed at Archangel.

### Discharged Neurasthenic Men.

Sir J. Craig, answering a question for the Pensions Minister, said that it is hoped that soon, for discharged men, a central neurological institution will be established in each region, available for both in-patient and out-patient treatment. It is recognised that in a great number of neurasthenic cases in-patient treatment is not required, and in order to provide for this a number of clinics are being established in various parts of the country where expert out-patient treatment will be given. It is expected that very soon ample accommodation will be available for the efficient treatment of these cases.

